# Metal-Free Click-Chemistry: A Powerful Tool for Fabricating Hydrogels for Biomedical Applications

**DOI:** 10.1021/acs.bioconjchem.4c00003

**Published:** 2024-03-22

**Authors:** Aysun Degirmenci, Rana Sanyal, Amitav Sanyal

**Affiliations:** † Department of Chemistry, Bogazici University, Bebek, Istanbul 34342, Türkiye; ‡ Center for Life Sciences and Technologies, Bogazici University, Bebek, Istanbul 34342, Türkiye

## Abstract

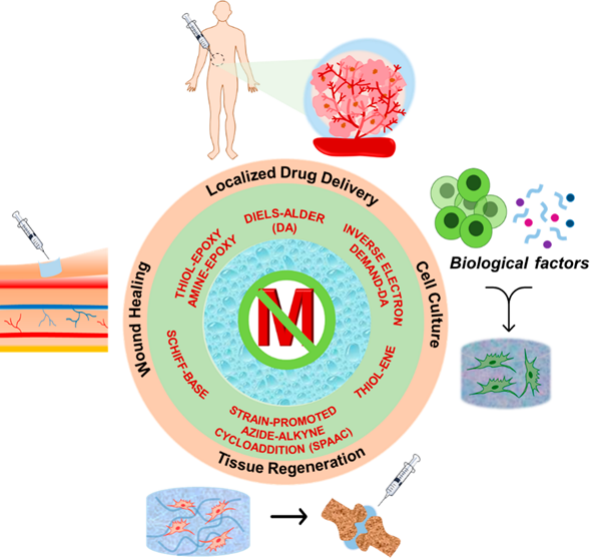

Increasing interest in the utilization of hydrogels in various areas of biomedical sciences ranging from biosensing and drug delivery to tissue engineering has necessitated the synthesis of these materials using efficient and benign chemical transformations. In this regard, the advent of “*click*” chemistry revolutionized the design of hydrogels and a range of efficient reactions was utilized to obtain hydrogels with increased control over their physicochemical properties. The ability to apply the “*click*” chemistry paradigm to both synthetic and natural polymers as hydrogel precursors further expanded the utility of this chemistry in network formation. In particular, the ability to integrate clickable handles at predetermined locations in polymeric components enables the formation of well-defined networks. Although, in the early years of “*click*” chemistry, the copper-catalyzed azide-alkyne cycloaddition was widely employed, recent years have focused on the use of metal-free “*click*” transformations, since residual metal impurities may interfere with or compromise the biological function of such materials. Furthermore, many of the non-metal-catalyzed “*click*” transformations enable the fabrication of injectable hydrogels, as well as the fabrication of microstructured gels using spatial and temporal control. This review article summarizes the recent advances in the fabrication of hydrogels using various metal-free “*click*” reactions and highlights the applications of thus obtained materials. One could envision that the use of these versatile metal-free “*click*” reactions would continue to revolutionize the design of functional hydrogels geared to address unmet needs in biomedical sciences.

## 1 Introduction

Hydrogels are three-dimensional polymeric networks that can retain a high amount of water and thus have found numerous biological applications in recent years.^1^ Over the past few decades, the role of these soft materials has evolved from static fluid reservoirs to dynamic materials that interact with their environment and thus play a functional role. In recent years, this class of network polymeric materials has gained wide attention due to their distinctive properties such as porous structure, biocompatibility, biodegradability, hydrophilicity, tunable mechanical properties, and ability to store and release small therapeutic agents to cells.^2,3^ Due to these aforementioned advantageous attributes, hydrogels have emerged as indispensable candidates for various biomedical applications such as drug delivery, biological sensing, and tissue engineering.^4−9^ Needless to say, for these materials to gain translational and pragmatic importance, the development of facile and practical methodologies for their fabrication is critical. Based on the nature of cross-linking, these soft materials can generally be categorized into two classes: physically and chemically cross-linked hydrogels. Physically cross-linked hydrogels may be obtained under mild conditions where polymer chain associations are governed through non-covalent interactions such as hydrogen bonding, electrostatic interactions, hydrophobic/hydrophilic interactions, host-guest chemistry, etc.^10−13^ However, physically cross-linked hydrogels often show poor mechanical properties due to the weak nature of these interactions. Chemically cross-linked hydrogels, on the other hand, are more stable and robust due to the presence of chemical bonds as interchain linkages. To date, general synthetic approaches toward hydrogel fabrication involve either simultaneous polymerization cross-linking of monomers or interchain cross-linking of polymers using appropriate chemical reactions. While both approaches have pros and cons, the latter is highly versatile, using natural or well-defined synthetic polymers as building blocks. The abundant availability, hydrophilic nature, and biocompatible nature of natural polymers make them attractive hydrogel precursors. Likewise, advances in contemporary polymer synthesis allow one to engineer various tailor-made polymers with control over their molecular weight, architecture, and placement of reactive groups. Thus, the fabrication of chemically cross-linked hydrogels using appropriately functionalized polymeric precursors has become a method of choice for synthesizing cross-linked materials for various biomedical applications.

Among various chemical transformations utilized to install interchain cross-linking, methods that proceed with high efficiency under mild conditions are in demand. Also, since these materials in many applications are intended for biological use, a cytocompatible nature is desirable. Depending on the application, hydrogels can be synthesized and then applied *in vivo* after proper procedures for removal of unreacted reagents and sterilization, or they can be employed as injectable forms. In particular, for the latter approach, it is of paramount importance that no toxicity arises from unreacted reactive groups on gel precursors, catalysts, and other reagents needed for the cross-linking or from byproducts generated during the cross-linking reaction. Furthermore, the high efficiency of the cross-linking reactions is vital to obtaining hydrogels with enough stability in the biological milieu. Another essential aspect in the case of injectable format is fast gelation kinetics in biological media to warrant the formation of a stable hydrogel at the site of application hydrogel. Since the advent of “*click*” chemistry,^14^ a set of chemical transformations which proceed with high efficiency under mild conditions and satisfy several other criteria such as selectivity and benign nature, including lack of formation of toxic byproducts, “*click*”-reaction-based methodologies for cross-linking polymers to obtain networks have gained momentum. We reviewed this area about a decade ago, on the tenth anniversary of “*click*” reactions.^15^ While reactions from the “*click*” toolbox continue to be employed for the synthesis of hydrogels, a survey of recent reports indicates a substantial shift toward employing metal-free “*click*” reactions.

For nearly the past two decades, several efficient “*click*” reactions have been employed to establish them as an effective tool for fabricating hydrogels and their subsequent functionalization. In particular, early efforts focused on the utilization of the Huisgen-type copper-catalyzed azide-alkyne cycloaddition (CuAAC) reaction.^16,17^ Since the early demonstrations of hydrogel synthesis using the CuAAC reaction by Hilborn and Hawker, separately in 2006,^18,19^ several studies have utilized these high-yield transformations to fabricate and functionalize hydrogels. While the rapid gelation and facile post-polymerization functionalization of hydrogels using the CuAAC reaction is highly attractive,^19−23^ using metal catalysts to fabricate hydrogels becomes a concern for utilization in biomedical fields since residual amounts of metal salt impurities may lead to compromise in the performance of these materials.^24^ While it is relatively easier to remove the copper-based catalysts for soluble polymers, warranting their removal from a cross-linked matrix where triazoles also are metal salt chelators presents a challenging scenario. In this regard, the fabrication of cross-linked materials without utilizing any metal-based catalyst presents a benign approach.

**Figure 1:**
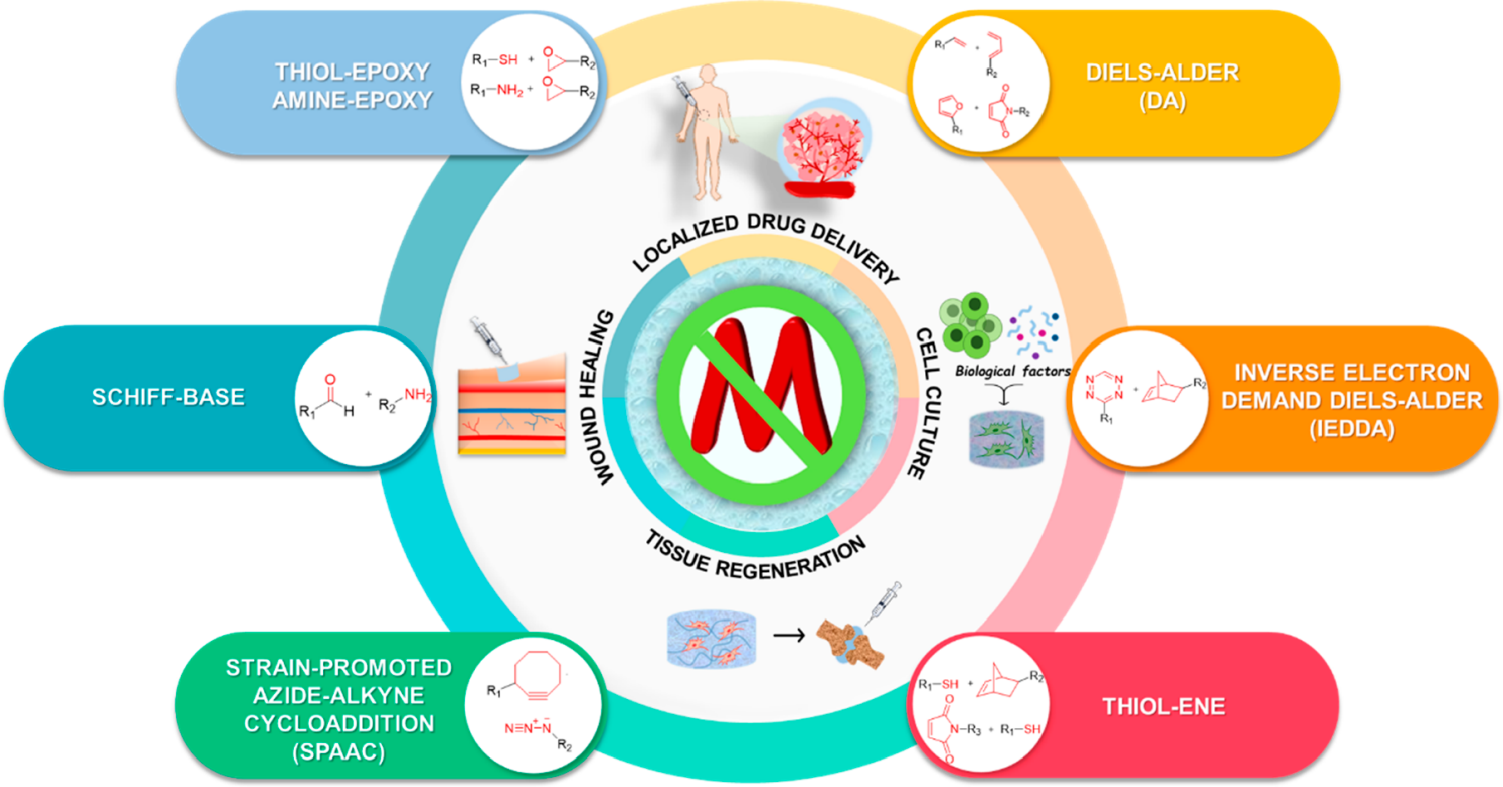
Illustration of commonly employed metal-free “*click*” reactions in fabricating hydrogels and related biomedical applications.

As an attractive alternative, metal-free “*click*” reactions have gained a lot of interest since many of these reactions also proceed with high reactivity and selectivity under mild reaction conditions.^25−27^ Indeed, a survey of recent literature on the design and application of biocompatible hydrogels shows that metal-free “*click*” reactions are emerging as a preferred method of choice. In this mini-review, we highlight the utilization of metal-free “*click*” reactions to fabricate hydrogels. We survey the commonly used metal-free “*click*” reactions such as the Diels-Alder (DA) and the inverse electron demand Diels-Alder (IEDDA) cycloaddition, thiol-ene radical addition, Michael type thiol-ene reactions, strain-promoted azide-alkyne cycloaddition (SPAAC), Schiff-base reaction, thiol-epoxy, and amine-epoxy “*click*” reactions (Table 1). This review aims to provide the reader with a quick overview of the use of these powerful metal-free reactions in the fabrication of hydrogels for biomedical applications (Figure 1).

**Table 1:**
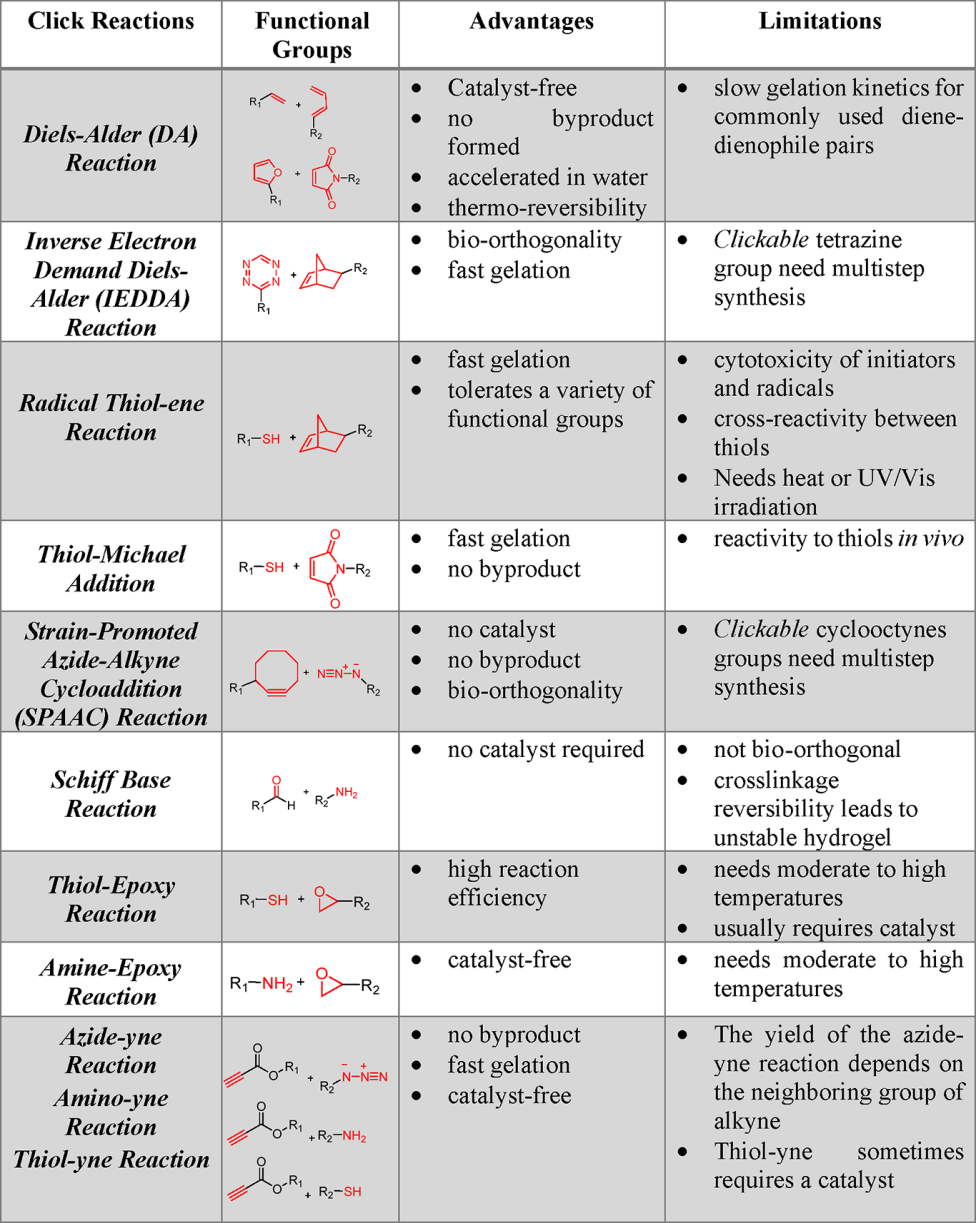
Survey of Commonly Used Metal-Free “*click*” Reactions, Advantages, and Limitations

## 2 Diels-Alder (DA) and Inverse Electron Demand Diels-Alder (IEDDA) Cycloaddition-Based Hydrogels

### 2.1 DA Cycloaddition-Based Hydrogels

Decades before the DA reaction was classified as a “*click*” reaction, hydrogel formation using this powerful cycloaddition reaction was reported by Chujo and co-workers in 1990, where they synthesized polyoxazoline-based thermally reversible hydrogels using cross-linking between furan and maleimide conjugated poly(*N*-acetylethylimine) polymers.^28^ Since this seminal report, although the DA reaction has been extensively employed to obtain thermally reversible cross-linked polymeric materials, most of the effort has focused on the fabrication of self-healing hydrophobic cross-linked polymeric materials. Classification of the DA reaction as a “*click*” reaction ignited a renewed interest in employing this reaction to fabricate hydrogels. Most of the studies utilize the furan-maleimide dyad since these reactive groups are readily available and cheap and can be easily integrated into natural and synthetic polymers. The DA reaction does not require any catalyst or initiator and does not lead to the release of toxic byproducts.^29^ While the furan-maleimide reaction proceeds at room temperature, it generally requires a long time. In this context, recent studies have shown that the rate of cycloaddition reaction could be accelerated by increasing the temperature and choosing a suitable solvent.^30,31^ More importantly, several reports demonstrate that the cycloaddition rate could be improved by using water as a solvent, which is desirable for fabricating hydrogels for biological applications.^32,33^ In light of these observations, there is an ever-increasing interest in utilizing the DA cycloaddition reaction to fabricate hydrogels.^34−59^ Some of these examples are briefly discussed to highlight the diverse hydrogels accessed by using this approach.

**Figure 2:**
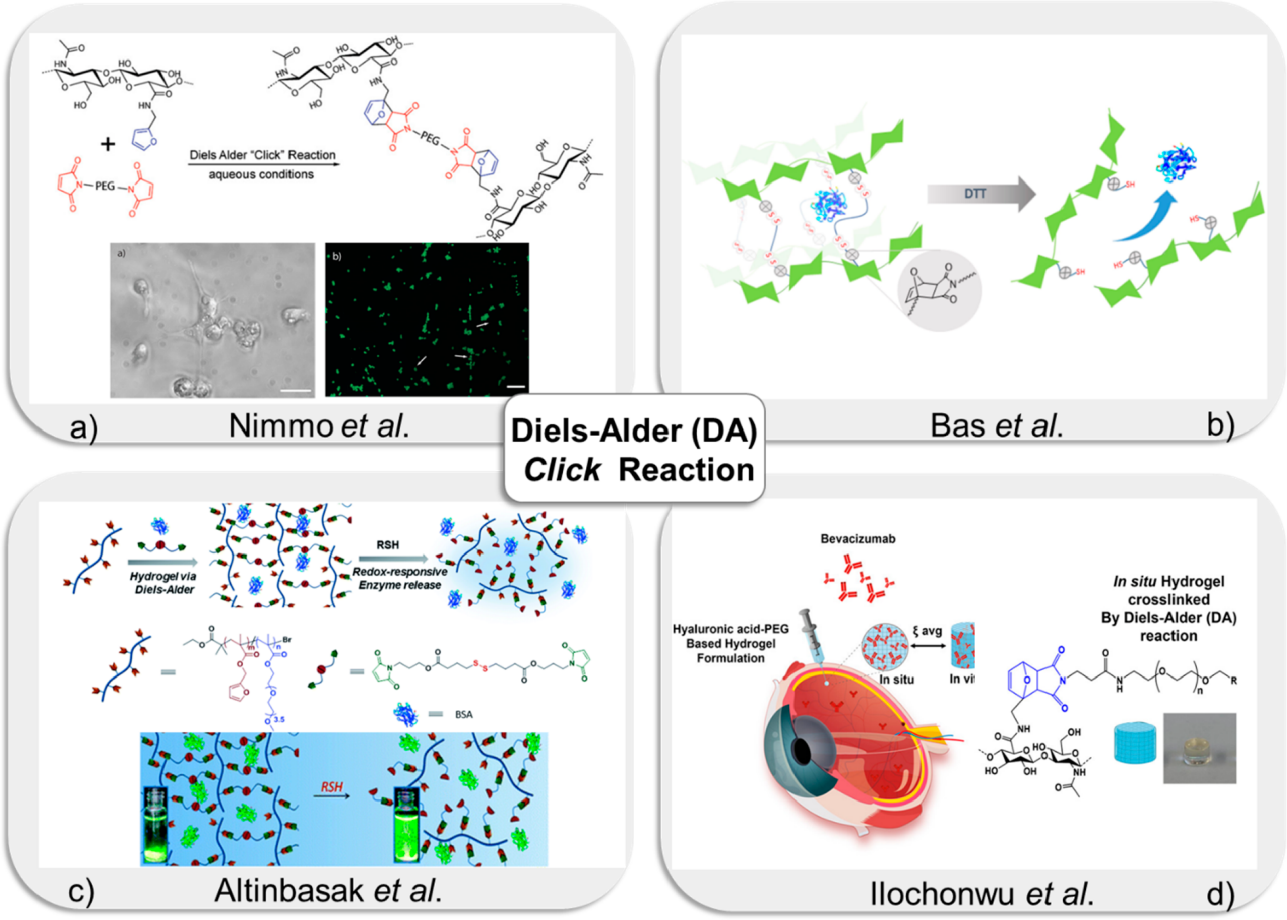
(a) Fabrication of hyaluronic acid (HA)-based hydrogels via the DA “*click*” reaction. Adapted with permission from Nimmo et al.^40^ Copyright 2011 American Chemical Society. (b) Fabrication of HA-based redox responsive hydrogels for release of biomacromolecules. Reprinted from Bas et al.^42^ with permission. Copyright 2023 Taylor and Francis. (c) Schematic representation of redox-responsive hydrogel via DA reaction for protein release. Adapted with permission from Altinbasak et al.^43^ Copyright 2016 The Royal Society of Chemistry. (d) Fabrication of hyaluronic acid-PEG-based DA hydrogels for delivery of bevacizumab. Adapted with permission from Ilochonwu et al.^56^ Copyright 2022 American Chemical Society.

Bai et al. fabricated hydrogels using a combination of non-covalent cross-linking via supramolecular interaction of cyclodextrin and adamantane with the thermosensitivity of poly(*N*-isopropylacrylamide) (PNIPAM) and chemical cross-linking furfuryl amine-grafted chondroitin sulfate (ChS-F) and maleimide-functionalized polyethylene glycol (PEG2K-AMI). Interestingly, the *in vivo* bone repair test demonstrated that the pure hydrogel could induce bone repair without using cells or growth factors.^39^ Another elegant utilization of the DA reaction was reported by Shoichet and co-workers for the fabrication of hyaluronic acid (HA)-based hydrogels for tissue engineering scaffolds (Figure 2a).^40^ HA-based hydrogels were obtained by cross-linking furan-modified HA with a telechelic bismaleimide-PEG. Obtained HA-PEG hydrogels displayed tunable mechanical and degradation properties, along with high cellular viability (>98%). In a related approach, Yu et al. reported the synthesis of the multifunctional hydrogel by combining the DA reaction with the aldehyde-amine Schiff-base reaction.^41^ A double cross-linked hydrogel was obtained by mixing aldehyde and furan-containing hyaluronic acid, adipic dihydrazide, furan-containing hyaluronic acid, and bis-maleimide PEG cross-linker. While the DA reaction maintained the structural integrity and mechanical property of hydrogel under physiological conditions, the acylhydrazone bond, forming after the Schiff-base reaction, resulted in imparting the self-healing property of these hydrogels. Authors envisioned that such hydrogels may find application in tissue engineering.

Although the reversible nature of the DA reaction between furan and maleimide is widely exploited as a reversible linkage in self-healing polymeric materials, its use in hydrogels has not been explored much. This is because the temperature required to break the furan-maleimide cycloadduct rapidly and effectively is higher than room temperature. This scenario is, in general, not suitable for biological applications. Accessibility to a trigger to break the cross-links is attractive since it could be exploited to release the cargo encapsulated within the hydrogels. In this context, Sanyal and co-workers reported the fabrication of redox-responsive hydrogels using the DA “*click*” reaction. Redox-responsive hydrogels were prepared using furan-containing HA and PEG-based disulfide-containing bis-maleimide-based cross-linker, to obtain on-demand release of the macromolecular cargo, FITC-BSA (Figure 2b).^42^ Disulfide bonds are known to undergo degradation in reducing environments, such as the presence of thiol-containing molecules like glutathione. The glutathione-based cleavage strategy has been widely utilized to obtain redox-responsive degradable polymeric networks.^60−64^ In another example, the same group obtained hydrogels using a furan-containing PEG-based hydrophilic copolymer and a disulfide-containing bis-maleimide-based cross-linker.^43^ They demonstrated that the obtained hydrogels could be completely degraded in a DTT solution. Degradation under a reducing environment enabled modulation of protein release (Figure 2c). On a long time scale, it is known that the *endo* isomers of the furan-maleimide adducts are slowly reversible to starting precursors at room temperature. This slow reversibility can be exploited to design a long-term protein-release hydrogel system, whereby the encapsulated biomolecule is released with time as the furan-maleimide cycloadducts undergo cleavage. Goepferich and co-workers reported examples of degradable hydrogels fabricated using the DA chemistry for the controlled release of therapeutics.^51−54^ They prepared degradable and thermosensitive hydrogels using maleimide and furan-modified 4- and 8-arm poloxamine via DA chemistry.^51^ In these hydrogels, more than 90% of bevacizumab was released and 87% of released bevacizumab showed binding ability. In a related approach, Vermonden and co-workers reported hyaluronic acid-PEG-based hydrogels fabricated using the DA reaction.^55,56^ They obtained stable hydrogels using hyaluronic acid-bearing furan groups (HAFU) and 4-arm-PEG10K-maleimide (4-APM).^56^ In these hydrogels, the release kinetics of encapsulated bevacizumab antibody was tunable by adjusting the ratio of the polymeric precursors, and a hydrogel sustained release over more than 400 days was achieved (Figure 2d).

Despite several attractive attributes of the DA cycloaddition reaction, slow reactivity has been one of the main concerns. One approach to address this problem is to utilize more electron-rich dienes. Such a strategy has been exploited by Shoichet and co-workers, who utilized methyl-furan, a diene with a highly electron-rich furan ring.^57^ Indeed, a methyl-furan substituted HA polymer upon mixing with bis-maleimide containing telechelic PEG polymers resulted in gelation within 12 ± 2 min at physiological pH. These hydrogels were shown to be suitable for encapsulation and 3D culture of cells. Another approach involves the use of electron-rich dienes, such as fulvenes. Li, Chen, and co-workers reported the synthesis of dextran-based self-healing hydrogels under physiological conditions by mixing fulvene-conjugated dextran with dichloromaleic acid-containing telechelic PEG-based cross-linker. The gelation time could be varied between 5 and 90 min, depending on the ratio of the dienophile to diene.^58^ Recently, a fulvene-maleimide cycloaddition reaction was reported by Madl and Heilshorn to obtain hydrogels with rapid gelation kinetics and high stability. Successful homogeneous encapsulation of mesenchymal stem cells was demonstrated for hydrogels obtained by mixing multiarm PEGs containing the fulvene and maleimide groups at their chain ends.^59^ The authors also extended the approach to obtain hybrid protein-synthetic polymer hydrogels by combining fulvene-conjugated elastin-like proteins with tetra-arm PEG maleimide.

### 2.2 IEDDA Cycloaddition-Based Hydrogels

Recently, the IEDAA “*click*” reaction has attracted significant attention due to the excellent orthogonality and biocompatibility of this system. The IEDDA reaction, first discovered by Bachmann and Deno in 1949, involves the reaction of an electron-rich dienophile with an electron-poor diene.^65^ In addition to bio-orthogonality and biocompatibility, the IEDDA reaction is fast, selective, and catalyst-free. Because of these attributes, in recent years, the IEDDA-reaction-based approaches have been explored in biomedical areas such as tissue engineering, drug delivery, biolabeling, and material science.^66−68^

**Figure 3:**
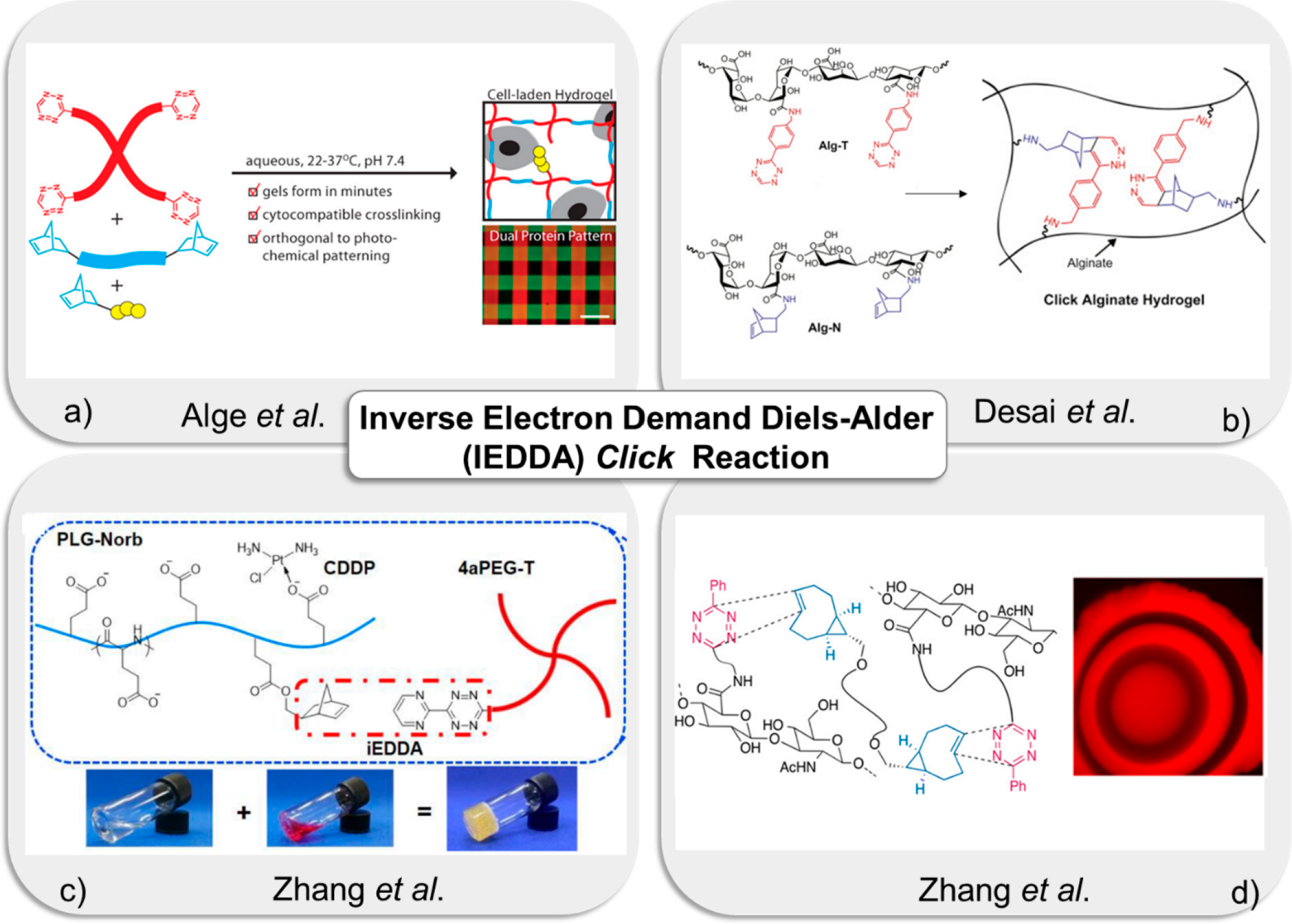
(a) Fabrication of tractable “*click*” hydrogels for three-dimensional cell culture. Adapted with permission from Alge et al.^69^ Copyright 2013 American Chemical Society. (b) Fabrication of alginate-based hydrogels via tetrazine-norbornene chemistry. Adapted with permission from Desai et al.^72^ Copyright 2015 Elsevier. (c) Schematic illustration of injectable “*click*” polypeptide hydrogels for local tumor treatment. Adapted with permission from Zhang et al.^78^ (d) Fabrication of interfacial bio-orthogonal cross-linking for 3D patterning and cell culture. Adapted with permission from Zhang et al.^82^ Copyright 2014 American Chemical Society.

The high yield and evolution of a benign byproduct (N_2_ gas) make the IEDDA reaction an ideal tool for the fabrication of biocompatible hydrogels, including injectable ones.^69−80^ In a seminal contribution, Anseth and co-workers utilized the tetrazine-norbornene IEDDA reaction to obtain PEG-based hydrogels suitable for protein patterning and cell encapsulation.^69^ A bis-norbornene-functionalized peptide was utilized as a cross-linker to obtain a cell-degradable scaffold. Human mesenchymal stem cell (hMSC) laden hydrogels were obtained by mixing the cells with a solution of the tetra-PEG macromonomer and peptide-based cross-linker susceptible to degradation by cell-secreted matrix metalloprotease (MMP) enzymes. To promote cell-matrix interactions, norbornene-RGDS was added to the gelation mixture. Using this strategy, a high level of post-encapsulation cell viability of 92 ± 2 and 79 ± 6 after 24 and 72 h, respectively, was achieved (Figure 3a). Shortly after, Joshi, Mooney, and co-workers utilized the tetrazine-norbornene chemistry to obtain alginate-based hydrogels.^72^ The residual norbornene units could be functionalized with thiol-containing peptides using the photochemical thiol-ene “*click*” reaction. The hydrogels were able to encapsulate cells with high viability, had minimal inflammatory response, and were stable *in vivo* for a longer time than their ionic counterparts obtained through calcium-mediated cross-linking (Figure 3b).

In addition to cell encapsulation and tissue engineering, IEDDA-based hydrogels can also be used for *in situ* drug and protein release.^77−81^ Famili and Rajagopal combined tetrazine appended hyaluronic acid and bisnorbornene-PEG to obtain Fab1 antibody fragment encapsulated hydrogels.^77^ The *in situ* encapsulated protein was released over a period of several days to weeks, depending upon the gelation concentration. The bio-orthogonal nature of the gel formation process ensured the retention of stability and binding activity of the released antibody. Chen and co-workers reported the fabrication of injectable polypeptide-hydrogel using an IEDDA “*click*” reaction for localized cisplatin release.^78^ This injectable hydrogel was obtained by mixing norbornene-modified poly(l-glutamic acid) (PLG-Norb) and tetrazine-functionalized four-arm poly(ethylene glycol) (4aPEG-T). Cisplatin was loaded into the hydrogel using polymer-metal complexation with the carboxylic acid groups of PLG-Norb. In an MCF-7-bearing mice model, this cisplatin-loaded polypeptide hydrogel exhibited an improved antitumor effect with reduced toxicity due to localized and sustained cisplatin release into the targeted region (Figure 3c). Lim and co-workers synthesized injectable and biocompatible alginate-based hydrogel via IEDDA “*click*” reaction for DOX release.^79^ A water-soluble and tetrazine-functionalized PEG-based cross-linker was synthesized for hydrogel synthesis, and disulfide linkages were introduced between PEG and tetrazine units to obtain a redox-responsive cross-linker (DTz-DS-PEG). The hydrogel was obtained using the IEDDA “*click*” reaction between the norbornene-functionalized alginate (Alg-Nb) polymer and DTz-DS-PEG cross-linker. The resulting hydrogels demonstrated high swelling ratios, porous morphology, and high DOX loading efficiency. *In vitro* drug release experiments revealed that hydrogels showed more DOX release (>90%) in the presence of glutathione (GSH, 10 mM) compared with PBS buffer (<25%). While empty hydrogel did not exhibit significant cytotoxicity toward fibroblast cells, DOX-loaded hydrogels induced a cytotoxic effect against cancer cells.

Another commonly used dienophile that is widely used with tetrazine (Tz) is the strained trans-cyclooctene (TCO) moiety.^82−88^ The first example of using a Tz-TCO dyad for gelation was reported by Fox, Jia, and co-workers.^82^ In this elegant work, microspheres were obtained using interfacial reaction when the Tz-functionalized hyaluronic acid (HA-Tz) was dropped into a bath of bis-TCO cross-linker. Due to the fast kinetics of the reaction, microspheres with core-cross-linked shells were obtained. Further treatment with non-fluorescently tagged TCO and fluorescent-dye-labeled TCO could diffuse into these microspheres to achieve spatially controlled labeling. To demonstrate the cytocompatible nature of the process, prostate cancer LNCaP cells were encapsulated within the microspheres formed upon the addition of the mixture of cells and HA-Tz into a bis-TCO cross-linker-containing bath (Figure 3d).

## 3 Thiol-ene Reaction-Based Hydrogels

### 3.1 Radical Thiol-ene Addition-Based Hydrogels

Radical thiol-ene “*click*” reaction between a thiol and an alkene group is a metal-free, efficient, high-yielding, fast reaction that is tolerant to a variety of functional groups.^89^ Due to these advantages, thiol-ene photo “*click*” reactions have been widely used to fabricate and functionalize hydrogels.^90−117^ The reaction can be initiated thermally or photochemically using the appropriate radical initiators. In a seminal contribution, an example of an enzyme-responsive biodegradable hydrogel fabricated via the thiol-ene “*click*” reaction was reported by Anseth and co-workers.^95^ A thiol-ene “*click*” reaction between norbornene-functionalized tetra-arm PEG and bis-cysteine human neutrophil elastase (HNE) sensitive peptide yielded degradable hydrogels suitable for protein-release applications.

**Figure 4:**
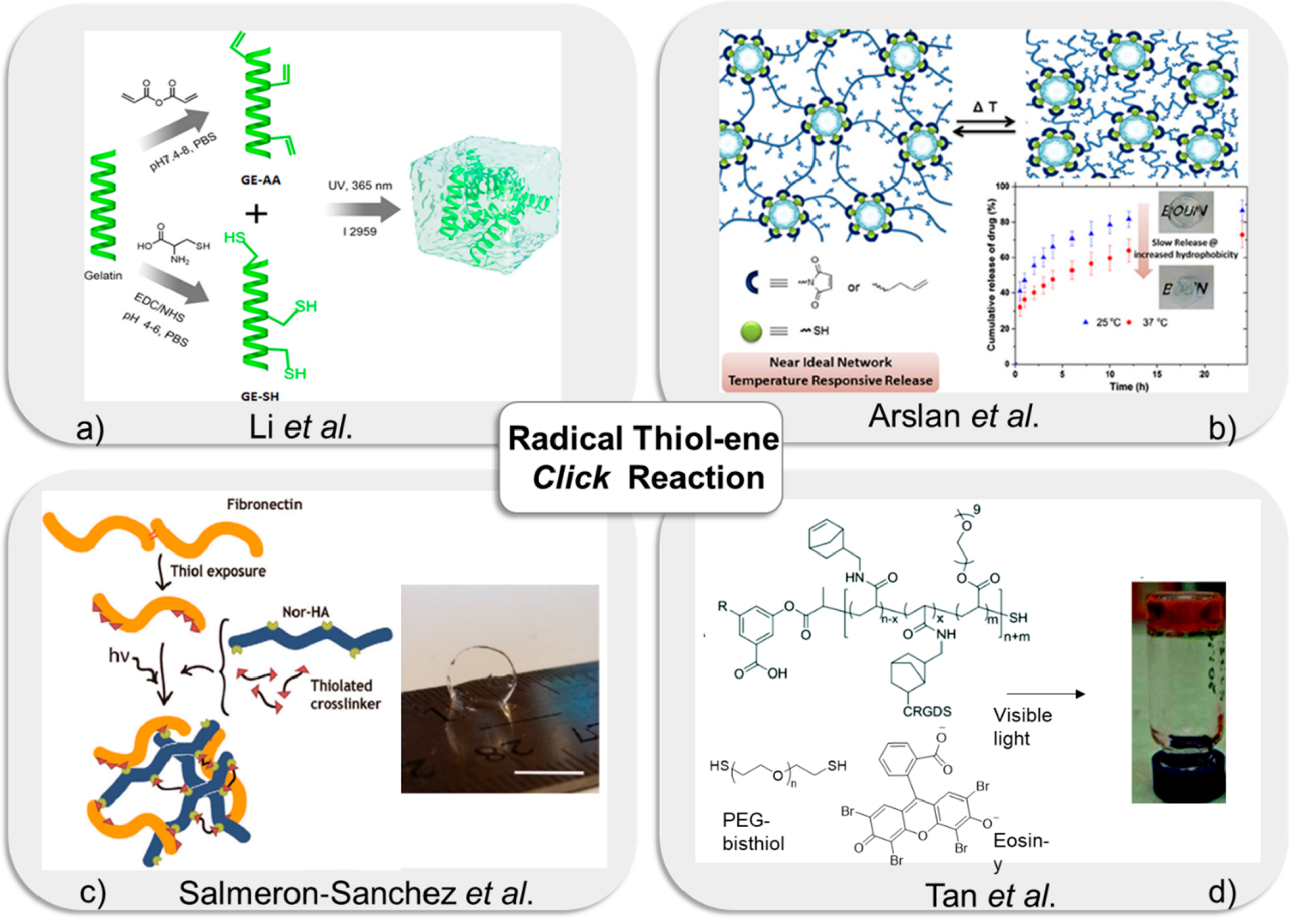
(a) Schematic synthesis of GE-AA and GE-SH and schematic description of the mechanism of network formation between GE-AA and GE. Adapted with permission from Li et al.^96^ Copyright 2018 American Chemical Society. (b) Illustration of thermoresponsive hydrogel fabrication using thiol-ene “*click*” reaction and their drug release profiles. Adapted from Arslan et al.^109^ Copyright 2017 American Chemical Society. (c) The schematic illustration of hyaluronic acid-fibronectin hydrogel via thiol-ene chemistry. Adapted from Salmeron-Sanchez et al.^113^ Copyright 2020 Wiley VCH GmbH. (d) The synthesis of photoinduced RAFT polymerized hydrogels for cell culture. Adapted from Tan et al.^115^ Copyright 2017 The Royal Society of Chemistry.

Hughes and co-workers reported the fabrication of gelatin-based injectable and photocurable hydrogels for corneal wound repair. Thiol-ene “*click*” reaction between acrylated gelatin (GE-AA) and thiolated gelatin (GE-SH) resulted in the formation of hydrogel with tunable mechanical, biodegradable, and biological properties. The obtained hydrogels demonstrated high cell viability and biocompatibility toward rabbit cornea, with no detrimental effect of UV irradiation on the cornea (Figure 4a).^96^ Using the thiol-ene reactions, Sanyal and co-workers have reported the fabrication of PEG-based hydrogels.^107−109^ In one of their studies, authors synthesized chemically cross-linked hydrogels using allyl-group-functionalized telechelic-PEG and heptavalent thiol-modified βCD. Water uptake capacity, morphologies, and rheological behavior of these hydrogels could be adjusted by changing the length of the PEG chain or by varying the amount of βCD-based cross-linker. The sustained release of a glaucoma drug, namely, puerarin, was demonstrated from these βCD-containing hydrogels.^108^ As an extension of this work, the authors reported the fabrication of βCD-containing thermoresponsive hydrogels using copolymers containing PEG-based side chains. Thiol-ene reactions between homotelechelic maleimide and vinyl-functionalized copolymers and a heptathiol-functionalized β-CD-based cross-linker yielded hydrogels with good conversions and tunable swelling and mechanical properties. The cytocompatibility of these hydrogels with respect to fibroblast cells was demonstrated. In addition, it was shown that a more sustained drug release was observed at physiological temperatures compared to that observed under ambient conditions (Figure 4b).^109^ Burdick and co-workers have investigated the fabrication of hydrogels using norbornene-thiol chemistry.^110−113^ In one of their studies, the authors synthesized fibronectin HA-based hydrogels using thiol-ene chemistry for stem cell engineering (Figure 4c).^113^ HA-based hydrogels were obtained using thiol-containing cross-linker and norbornene-functionalized HA, and fibronectin was introduced to hydrogel during UV-triggered thiol-ene cross-linking. They showed that this approach enables the encapsulation proliferation of cells.

While the UV-initiated thiol-ene “*click*” reaction is an efficient method to fabricate hydrogels, utilization of a catalyst and UV light may restrict its application. Therefore, efforts have been devoted to fabricating hydrogels using visible or IR-mediated thiol-ene reaction.^103,114−116^ Myung and co-workers reported an *in situ* forming hyaluronic acid-based hydrogel via visible-light-induced thiol-ene reaction.^103^ The HA-based hydrogel was obtained through a thiol-ene reaction between methacrylated-HA and thiolated HA in the presence of visible blue light and riboflavin phosphate (RFP, vitamin B2) as an initiator. They demonstrated that the ability of blue light to initiate a reaction was equal to or more effective than that of UV light. Undertaking the reaction in visible light and with a biocompatible photoinitiator makes this hydrogel system promising for local drug delivery applications. Gooding and co-workers reported hydrogel formation via visible-light-induced thiol-ene reaction for 3D cell encapsulation.^115^ Norbornene-functionalized poly(ethylene glycol)methyl ether acrylate (PEGMEA)-based copolymer was prepared using RAFT polymerization. Utilization of PEG-bisthiol as a cross-linker yielded hydrogels through a thiol-ene reaction when visible light and eosin-Y were used to induce the reaction. This hydrogel system showed low cytotoxicity against encapsulated pancreatic cancer cells, and the conjugation of CRGDS peptide onto the hydrogel improved cell adhesion; however, cells did not show spreading (Figure 4d).

### 3.2 Thiol-ene Michael-Addition-Based Hydrogels

Fabrication of hydrogels using the thiol-Michael addition reaction, which occurs between thiol and the electron-deficient C=C bond, provides an attractive approach due to its high reactivity under relatively mild reaction conditions. Alkene groups bearing electron-withdrawing units such as ester, amide, and cyano functional groups are susceptible to base/nucleophile mediated thiol-Michael addition.^118^ The system’s reactivity largely depends on the nature of the electrophilic alkene unit. Bowman and co-workers observed the C=C bond reactivity order as propyl maleimide > diethyl fumarate > diethyl maleate > dimethyl acrylamide > acrylonitrile > ethyl crotonate > ethyl cinnamate > ethyl methacrylate when hexanethiol was employed as a nucleophile in the presence of hexylamine as a catalyst.^119^ Due to the high reactivity of the thiol group (especially in the presence of a mild organobase) and the ready availability or facile introduction of the reactive counterparts into macromolecular constructs, a variety of cross-linked materials, including hydrogels, have been prepared using the thiol-Michael addition.^120−155^

**Figure 5:**
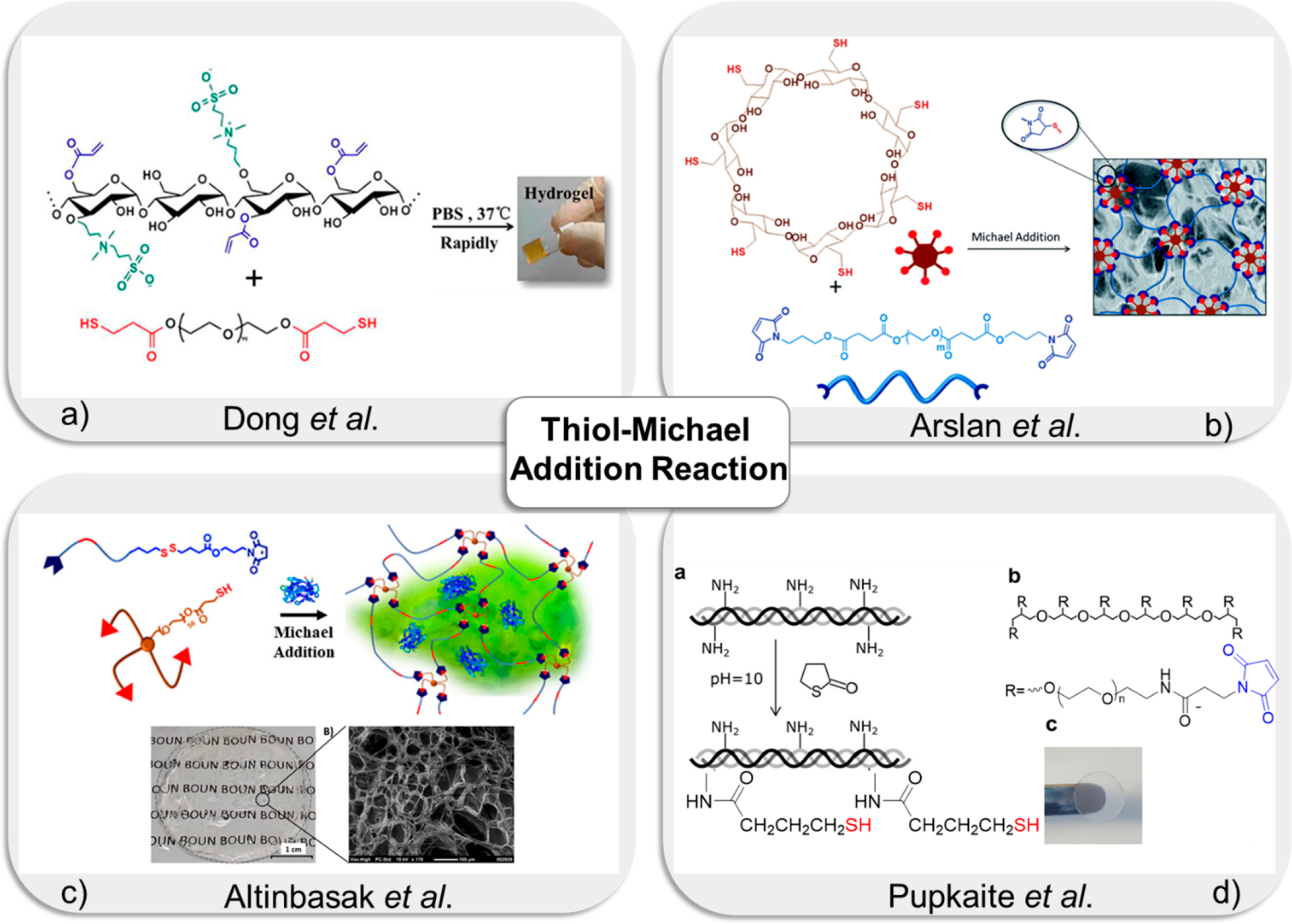
(a) Fabrication of zwitterionic starch-based hydrogel via thiol-Michael addition “*click*” reaction for cell encapsulation. Adapted with permission from Dong et al.^129^ Copyright 2016 American Chemical Society. (b) Schematic illustration of the fabrication of hydrogels via thiol-maleimide conjugation. Adapted with permission from Arslan et al.^134^ Copyright 2014 The Royal Society of Chemistry. (c) Synthesis of redox-responsive hydrogel using thiol-maleimide addition. Adapted with permission from Altinbasak et al.^135^ Copyright 2022 American Chemical Society. (d) Fabrication of injectable collagen hydrogel via thiol-Michael addition “*click*” reaction for cell encapsulation. Adapted with permission from Pupkaite et al.^144^ Copyright 2019 American Chemical Society.

As one of the early reports, the potential of the thiol-Michael addition reaction for obtaining hydrogels was evaluated by Hubbell and Rizzi.^120^ The authors obtained a recombinant protein polymer hybrid network by reacting thiol-containing protein-polymer conjugates with divinyl sulfone PEG under physiological conditions. The recombinant protein was designed to obtain materials incorporating key features of the extracellular matrix. Another early work that reports an elegant utilization of the thiol-Michael reaction was reported by Kopeček and co-workers for obtaining a protein-embedded hydrogel.^121^ A mutant enzyme incorporating two thiol groups was mixed with maleimide-containing synthetic polymers to obtain hydrogels that exhibited a volume change upon substrate recognition. More recently, the thiol-Michael addition was employed by Yao and co-workers who prepared *in situ* clickable zwitterionic starch-based hydrogels for cell encapsulation (Figure 5a).^129^ Hydrogel was obtained using acrylate, sulfobetaine-derived starch (SB-ST-A), and dithiol-modified PEG under physiological conditions. The authors demonstrated that hydrogel surfaces could resist nonspecific protein and cell adhesion. Furthermore, when A549 lung cancer cells were encapsulated in the hydrogel, they maintained 93% of their viability. In a clever approach, Theato and co-workers reported the fabrication of self-healing hydrogels via one-pot thiol-ene “*click*” and borax-diol chemistry.^130^ They prepared a hydrogel using commercially available poly(ethylene glycol)diacrylate, dithiothreitol, and borax. Borax played a dual role; it catalyzed the thiol-Michael addition reaction and acted as a cross-linker for the polymers obtained by step-growth polymerization. Authors demonstrated that hydrogels were self-healable, pH-responsive, and thermoresponsive due to the presence of boronate ester-based cross-links.

The high efficiency of the thiol-maleimide coupling reaction enables obtaining well-defined hydrogels using a multifunctional cross-linker. In this regard, Sanyal and co-workers reported the fabrication of cyclodextrin-containing PEG-based hydrogels via the thiol-maleimide reaction (Figure 5b).^134^ Maleimide-containing telechelic PEGs with different molecular weights were synthesized, and hydrogels were obtained with high efficiency in the presence of a catalytic amount of triethylamine when maleimide-containing polymers were reacted with thiolated β-cyclodextrin (β-CD(SH)_7_). Tuning the stoichiometry of the reactive functional groups allows one to obtain hydrogels that can undergo post-gelation functionalization using subsequent Michael addition. The conjugation of fluorescent dye molecules demonstrated the effective functionalization of these hydrogels. One limitation of the thiol-Michael addition approach is that these bonds are generally not reversible. As recently reported by Sanyal and co-workers, this can be addressed by judicious design of linkers. Redox-responsive degradable hydrogels were fabricated using a combination of thiol-maleimide conjugation and thiol-disulfide exchange reaction (Figure 5c).^135^ Maleimide-disulfide terminated telechelic linear PEG and PEG-based tetrathiol macromonomer were employed as gel precursors. Encapsulation and release of fluorescent-dye-labeled dextran and BSA protein from hydrogels suggested that, while passive release could be controlled using the molecular weight of the precursors, on-demand rapid release could be obtained upon exposure to a reducing environment. Thus, while the thiol-maleimide coupling ensures rapid gelation, the thiol-disulfide exchange reaction enables the dissolution of the hydrogel.

Even though hydrogels have been used as platforms for localized drug delivery, several challenges remain to be addressed, one of them being rapid and burst release. An approach that enables more sustained delivery involves conjugating the drug onto the hydrogel scaffold rather than physical encapsulation of the drug. This minimizes burst release as well as enables long-term controlled release of the active ingredient. For such purposes, Gao et al. fabricated an injectable hydrogel using the thiol-Michael addition reaction.^140^ Poly(oligo(ethylene glycol)maleate) (POEGM) copolymer was synthesized by using condensation polymerization of PEG and maleic anhydride in the presence of Sc(OTf)_3_ as a catalyst. The obtained copolymer had several electron-deficient alkene groups along the backbone and hydroxyl groups at the chain termini. An anticancer drug, camptothecin (CPT), was conjugated to the chain ends through a carbonate linkage. Hydrogel was obtained upon mixing this drug-containing copolymer with polyoligo(ethylene glycol) mercaptosuccinate (POEGMS), a copolymer containing multiple thiol functional groups. The authors demonstrated that the CPT-containing hydrogels showed significant *in vitro* cytotoxicity against HepG2 cells.

Apart from the delivery of drugs,^140−142^ hydrogels obtained using the thiol-Michael addition can be loaded with cells for their localized delivery.^143−153^ Samanta and co-workers reported an example of a self-healing injectable hydrogel, which involves thiol-maleimide-based covalent cross-linking. An injectable hydrogel with excellent shear-thinning and self-healing properties was obtained using thiol-containing collagen and maleimide-functionalized 8-arm PEG, without the need for any catalyst (Figure 5d).^144^ The authors also demonstrated that the hydrogel was cytocompatible and suitable for cell delivery in regenerative medicine and tissue engineering. Additionally, the authors noted that the hydrogels did not show any swelling in aqueous media. To benefit from this attribute, in a recent study, they employed a similar approach for obtaining sealants for corneal perforations.^145^ In another study, Wang and co-workers reported hyaluronic acid-based injectable hydrogel for delivery of cartilage-derived progenitor cells (CPCs).^152^ The hydrogel was obtained by cross-linking thiol-functionalized hyaluronic acid and a multiacrylated PEG-based macromonomer. Cells encapsulated within the hydrogel during the gelation process exhibited high viability and proliferation. It was observed that the CPC-loaded injectable hydrogel system could accelerate extracellular matrix (ECM) production and downregulate inflammation-related gene expression and thus may provide new approaches for cartilage regeneration.

## 4 Strain-Promoted Azide-Alkyne Cycloaddition-Based Hydrogels

As mentioned in the introductory section, among all reported “*click*” reactions, the CuAAC, which was discovered independently by Sharpless and Meldal,^14,16^ has drawn the most attention due to its high reaction efficiency under mild conditions, regioselectivity, and chemical orthogonality.^156−158^ Furthermore, fairly straightforward methods to incorporate azide and alkyne functional groups into polymeric precursors encouraged their utilization in synthesizing novel polymeric materials.^159−163^ Thus, the CuAAC reaction was extensively exploited to fabricate hydrogels soon after its discovery.^19−22,164^ Despite the attractive attributes, the CuAAC reaction may not be suitable for many biomedical applications. In instances where the complete removal of copper salts may not be possible, the remaining metal impurity could exhibit cytotoxic effects.^24,165^

A cycloaddition reaction between phenyl azide and a strained alkyne, namely, cyclooctyne, was reported by Wittig and Krebs in 1961.^166^ This metal-free azide-alkyne cycloaddition was popularized by Bertozzi and co-workers in 2004, who used it for chemical modification of living cells.^167^ The reaction has a low activation energy due to the cyclooctyne moiety’s high ring strain.^167^ In addition to the low activation energy, the SPAAC reaction does not require any catalyst and proceeds without forming any byproduct; therefore, this reaction possesses excellent biocompatibility. In recent years, due to its excellent biocompatibility, the SPAAC reaction has been used for the modification of surfaces of cells and viruses.^168,169^ These desirable features of the SPAAC reaction have led to a flurry of investigations reporting the fabrication of biocompatible hydrogels, many of them as injectable formulations.^170−188^

**Figure 6:**
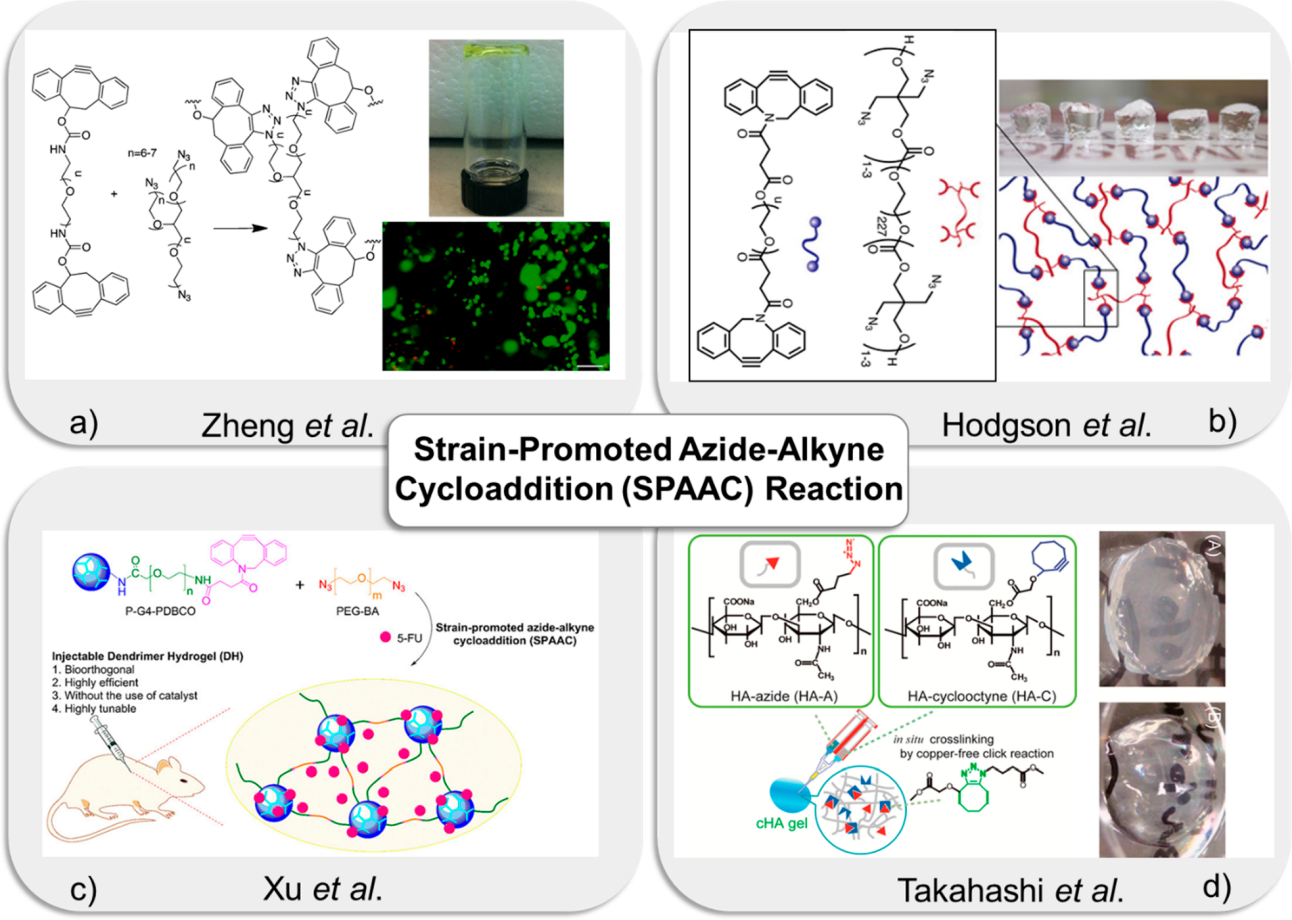
(a) Illustration of biocompatible PEG-based hydrogels via SPAAC cross-linking. Reprinted with permission from Zheng et al.^178^ Copyright 2012 American Chemical Society. (b) Illustration of cytocompatible PEG hydrogels via SPAAC reaction between aza-dibenzocyclooctyne (DIBAC) and azide. Reprinted with permission from Hodgson et al.^186^ Copyright 2016 American Chemical Society. (c) Representation of the drug-loaded dendron hydrogel system (5-FU/DH) *in vivo* by “SPAAC” “*click*” reaction. Reprinted with permission from Xu et al.^189^ Copyright 2017 American Chemical Society. (d) Schematic diagram of *in situ* cross-linking hydrogels of hyaluronan by copper-free “*click*” chemistry (cHA hydrogel) and physical appearances of cHA hydrogel. (A) Initial hydrogels before incubation and (B) 1 week after starting incubation. Adapted with permission from Takahashi et al.^191^ Copyright 2013 American Chemical Society.

The first report of utilization of SPAAC-based fabrication of hydrogels encapsulating cells was reported by DeForest et al.^176^ An enzymatically degradable hydrogel platform was created using gelation of a PEG tetra-azide polymer with a bis(difluorinated-cyclooctyne)-functionalized peptide cross-linker in an aqueous environment. Interestingly, the cross-linker was designed with an allyl group, which enabled post-gelation attachment of bioactive peptides using radical thiol-ene *click* reaction. In particular, a difluorescein collagenase-sensitive peptide was conjugated to the hydrogel. A localized increase in the fluorescence that occurs upon the cleavage of the peptide suggests high collagenase activity near the cell surfaces. The work thus highlights the power of the orthogonal nature of click reactions to obtain multifunctional hydrogels. Zheng et al. reported the synthesis of a PEG-based hydrogel using the copper-free SPAAC cross-linking strategy. They combined dibenzocyclooctynol-PEG (DIBO-PEG) and glycerol exytholate triazide to obtain a biocompatible hydrogel (Figure 6a).^178^ Hodgson et al. synthesized PEG-based hydrogels via the SPAAC reaction using aza-dibenzocyclooctyne terminated PEG polymer and azide precursors.^186^ By changing the ratio and concentration of the polymers, hydrogels with control over gelation time (from 10 to 60 s) and Young’s modulus (1–18 kPa) could be obtained. The PEG-based hydrogels showed minimal BSA protein adsorption and did not exhibit any cytotoxicity toward fibroblast cells (Figure 6b). The same research group also synthesized a dendronized PEG-based hydrogel via the SPAAC reaction. They obtained hydrogels using azide-terminated first- and second-generation (G1-PEG and G2-PEG) dendrons and dibenzocyclooctyne (DBCO)-based PEG. These polymer solutions gave hydrogels at low polymer concentrations with gelation times ranging from 10 s to 3.5 min. Hydrogels encapsulated with primary human mesenchymal stem cells (hMSCs) showed high viability over 2 weeks.^187^ Ono et al. synthesized biodegradable PEG-based hydrogel using the SPAAC reaction to encapsulate drug-loaded nanoparticles for drug release application.^188^ Two sets of ABA triblock copolymers, one composed of azide-containing outer blocks and the other with DBCO-containing outer blocks and PEG as an inner block, were mixed in the presence of Dox-loaded micelles to obtain a drug-encapsulated micelle-hydrogel composite. The drug release from the hydrogel matrix was more sustained than that observed for the micelle solution. Obtained drug-loaded micelle/hydrogel composite showed high cytotoxicity against MDA-MB-231 breast cancer cells. In another study, Xu et al. prepared dendrimer-based hydrogels (DH) through the SPAAC reaction between a polyamidoamine (PAMAM) dendrimer (G4) conjugated with DBCO using a PEG spacer (*M*_*n*_ = 2000 g/mol) and azide-containing telechelic PEG (*M*_*n*_ = 20,000 g/mol). Anticancer drug 5-FU was loaded into the hydrogel, and the DH/5-FU formulation significantly suppressed tumor growth and improved the survival of tumor-bearing mice (Figure 6c).^189^

Besides PEG-based hydrogels, natural polymers have also been used to fabricate hydrogels via the SPAAC “*click*” reaction.^190−193^ Takahashi et al. reported the synthesis of hyaluronic acid-based injectable hydrogel using hyaluronic acid modified with azide (HA-A) and cyclooctyne (HA-C). Hydrogel was obtained upon mixing these two polymers using a double-barreled syringe. The hydrogels degraded in 2 weeks, 9 days, and 4 days in PBS, hyaluronidase, and cell culture media with fetal bovine serum, respectively. Hydrogels were administered to mice subcutaneously and intraperitoneally. Depending on the administration method, the clearance time of the hydrogel residue changed from 7 to 21 days (Figure 6d).^191^ Wang et al. synthesized injectable dextran-based hydrogel using SPAAC reaction for cartilage tissue engineering. Injectable hydrogels based on dextran (Dex, *Mw* = 10,000 g/mol) were obtained under physiological conditions using azadibenzocyclooctyne-functionalized dextran (Dex-ADIBO) and azide-modified dextran (Dex-N_3_). It was observed that the gelation time of these hydrogels was dependent upon polymer concentrations and the ADIBO substitution degree on dextran. Afterward, rabbit chondrocytes were encapsulated within these hydrogels and employed to produce cartilage matrixes.^192^

## 5 Other “*Click*”-Reaction-Based Hydrogels and Their Applications

### 5.1 Schiff-Base-Linkage-Based Hydrogels

In recent years, the Schiff base reaction, an important “*click*” reaction, has attracted significant attention to fabricating self-healing hydrogels due to its facile nature, reversibility, pH-responsive property, and biodegradability.^194−199^ Since many biopolymers contain amine groups, the reaction has been widely employed to fabricate biodegradable hydrogels from natural polymers such as dextran, hyaluronic acid, and alginate.^195^ Schiff base linkers, consisting of imines, hydrazones, and oximes, are the products of condensation reactions between aldehyde and amine functional groups, which form without the use of any metal catalyst.^200^ However, the aldehyde group may react with the amine groups on the cell surface and other plasma proteins in the body; therefore, this reaction is not bio-orthogonal. The pH responsiveness of the linkage can be modulated as oximes and hydrazones show better chemical stability toward pH changes than imines. Schiff-base-reaction-based hydrogels have been evaluated in biomedical applications such as drug delivery,^201,202^ tissue regeneration,^203^ and wound healing.^204^ The reversible linkage imparts self-repair capability to hydrogels, which may be vital for hydrogels that suffer deformation-induced damage.^205−207^

**Figure 7:**
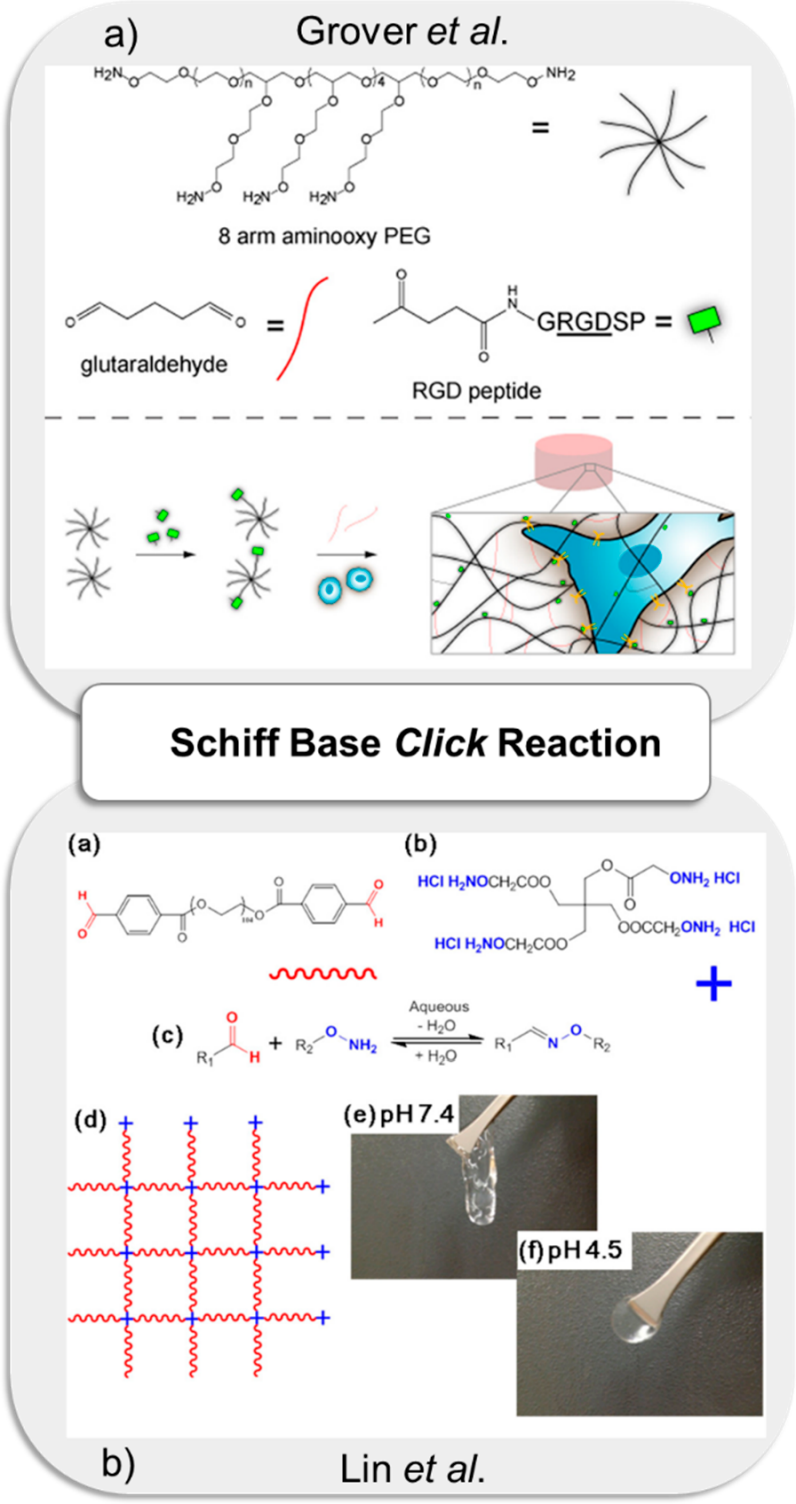
(a) Schematic illustration of RGD-functionalized PEG-based hydrogels via oxime “*click*” reaction for encapsulation of MSCs. Adapted from Grover et al.^198^ with permission. Copyright 2012 American Chemical Society. (b) Fabrication of peptide-functionalized oxime hydrogels. Adapted from Lin et al.^199^ with permission. Copyright 2013 American Chemical Society.

One of the earliest examples of Schiff-base hydrogel was reported by Hoffman and co-workers,^201^ who synthesized a PEG-based degradable hydrogel. Doxorubicin (Dox) was conjugated to the hydrogel with Schiff base bonds. The release study showed that the DOX release was pH-dependent. Roh and co-workers reported a fast-forming alginate-based hydrogel using an oxime-based “*click*” reaction. The study demonstrated that stress relaxation and mechanical properties of hydrogels could be tuned by changing the concentration of polymers and environmental factors like pH, temperature, and the use of catalyst. The biocompatibility of alginate-based hydrogels was demonstrated by encapsulating B-cells into a hydrogel matrix.^197^ Another interesting study was reported by Maynard and co-workers, who employed the oxime-based reaction for obtaining PEG-based biocompatible hydrogels by mixing 8-arm aminooxy PEG and glutaraldehyde. The study established that the mechanical properties of hydrogel could be tunable by changing the weight percent of the aminooxy-PEG and the ratio between aldehyde and amine groups. The RGD-peptide appended hydrogels supported mesenchymal stem cell (MSC) incorporation and high cell viability and proliferation, which displayed the biocompatible nature of the hydrogel (Figure 7a).^198^ In another study, Becker and co-workers reported the fabrication of peptide-functionalized oxime-based hydrogel. The gelation duration and hydrogels’ mechanical strength were tunable with pH and catalyst concentration (Figure 7b).^199^ The Schiff base reaction was also employed to obtain hydrogels composed of different biopolymers. For example, Ito and co-workers demonstrated the fabrication of an injectable gelatin-hyaluronic acid cross-linked hydrogel with slow degradability.^207^ Hydrogels were obtained by mixing carbohydrazide-functionalized gelatin (Gel-CDH) and mono aldehyde functional hyaluronic acid (HA-mCHO). Thus, the obtained biocompatible hydrogels underwent slow degradation in PBS buffer; hence, the hydrogels would be expected to be stable during angiogenesis, thus making them promising materials for tissue engineering applications.

### 5.2 Thiol-Epoxy and Amine-Epoxy-Based Hydrogels

**Figure 8:**
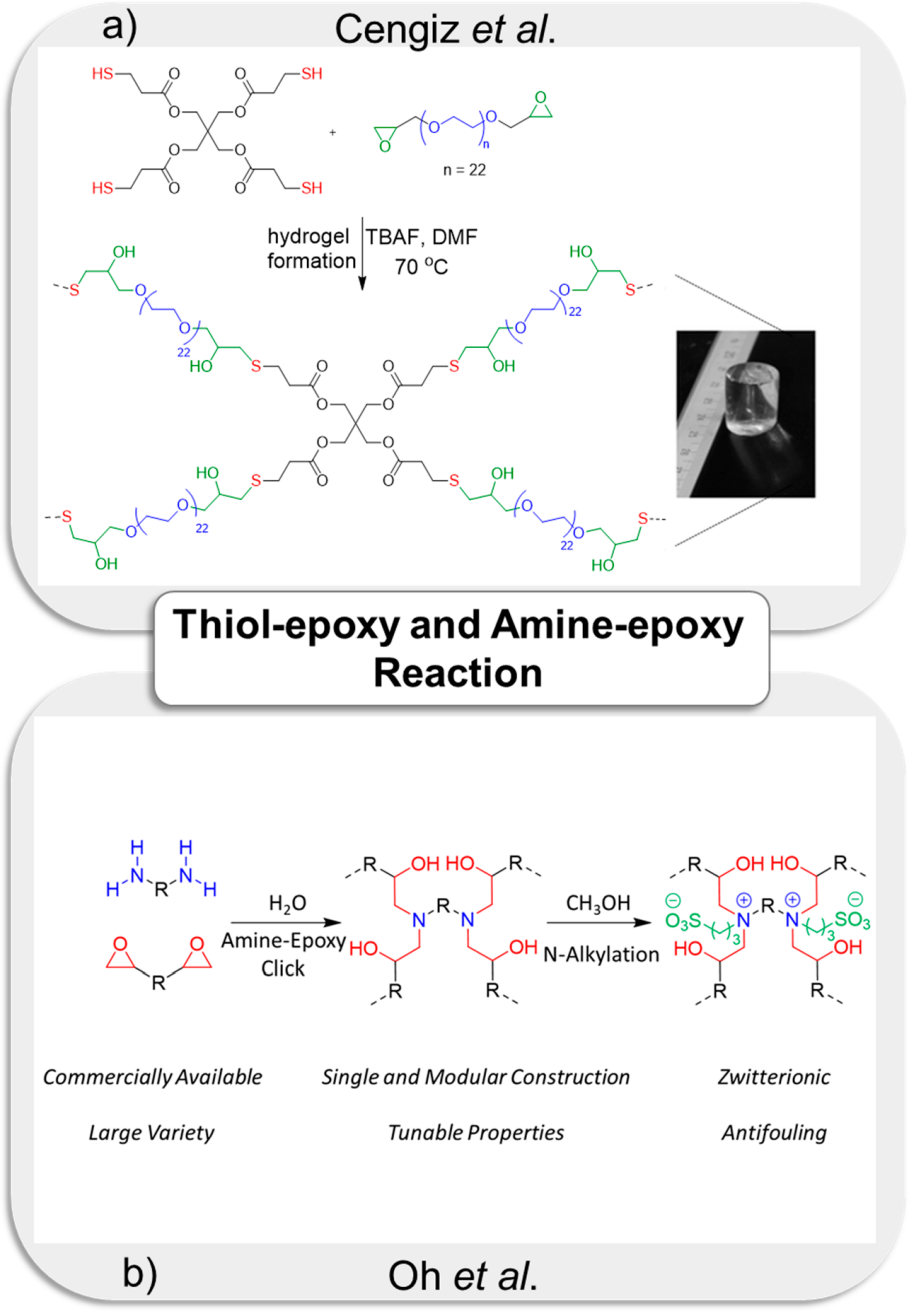
(a) The synthesis of functionalizable hydrogel using thiol-epoxy “*click*” reaction. Reprinted with permission from Cengiz et al.^211^ Copyright 2013 The Royal Society of Chemistry. (b) The fabrication of zwitterionic hydrogels via amine-epoxy “*click*” chemistry and *N*-alkylation reaction. Reprinted with permission from Oh et al.^218^ Copyright 2019 by the authors. Licensee MDPI, Basel, Switzerland.

In recent years, it has been demonstrated that the thiol-epoxy “*click*” reaction is an efficient transformation for synthesizing organic molecules, functional polymers, and polymeric materials.^208−210^ The reaction has been used to synthesize hydrogels using organic solvents^211^ and aqueous media.^212^ The thiol-epoxy “*click*” reaction offers many advantages for fabricating hydrogels, such as the ready availability of hydrogel precursors, high reaction efficiency, and tunable gelation time.^213^ Usually the reaction requires a base as a catalyst, which may make this approach difficult to adapt for injectable hydrogels.^211^ Khan, Sanyal, and co-workers reported the fabrication of functionalizable hydrogel using a thiol-epoxy reaction.^211^ Pentaerythritol tetrakis(3-mercaptopropionate) (PETMP) and glycidyl-functionalized telechelic PEG yielded hydrogels upon heating at 70 °C, in the presence of tetra-*n*-butylammonium fluoride (TBAF) as a catalyst (Figure 8a). Subsequent functionalization of newly formed hydroxyl groups in the obtained hydrogels was demonstrated through the attachment of a fluorescent dye, namely, 1-pyrene carboxylic acid. In addition, over the years, Khan and co-workers have reported several examples of hydrogel formation using this reaction.^213,214^ In another study, Alsberg and co-workers reported cytocompatible PEG-based hydrogel for cell encapsulation using a thiol-epoxy “*click*” reaction. They fabricated a fast-forming hydrogel by mixing solutions of eight-arm thiol-functionalized PEG (PEG(-SH)_8_) and diepoxy-containing PEG (PEG-DE) in basic aqueous media. Human mesenchymal stem cells (hMSCs) were encapsulated into the hydrogels, and cells demonstrated high viability over 4 weeks. It was noted that a pro-osteogenic siRNA-loaded hydrogel significantly promoted the osteogenic differentiation of hMSCs.^215^

In addition to the thiol-epoxy chemistry, the amine-epoxy chemistry is another “*click*”-based reaction because it does not require using a catalyst.^216^ Morrin and co-workers reported the fabrication of glucose-sensitive hydrogel using an amine-epoxy “*click*” reaction between aliphatic diamine and poly(ethylene glycol) diglycidyl ether (PEGDGE).^217^ They used this hydrogel system to detect glucose by entrapping glucose oxidase (GOx). In another example, Khan and co-workers reported the synthesis of zwitterionic hydrogels via amine-epoxy “*click*” chemistry and *N*-alkylation reaction (Figure 8b).^218^ They prepared a hydrogel using commercially available PEG-diamine and PEG-epoxy precursors in aqueous media without any catalyst. The resulting hydrogel was subjected to an alkylated ring-opening reaction to obtain zwitterionic materials. In an elegant study, the amine-epoxy chemistry has been combined with redox-responsive linkages by Cengiz,^219^ who reported glutathione-responsive hydrogels. Hydrogels were obtained using PEG-diamine and disulfide-containing redox-responsive diepoxy cross-linker under catalyst-free conditions. Newly formed hydroxyl groups and residual epoxides within the hydrogel were utilized for their functionalization with biomolecules and fluorescent dyes. The orthogonal nature of the amine-epoxy coupling chemistry with other types of “*click*” reactions can be used to obtain dual-network hydrogels, as reported by Hawker and co-workers.^220^ The amine-epoxy chemistry has been used in conjunction with the CuAAC chemistry to obtain dual-cross-linked hydrogels which were highly robust and showed excellent mechanical properties.

### 5.3 Azide-yne, Amino-yne, and Thiol-yne Reaction-Based Hydrogels

**Figure 9:**
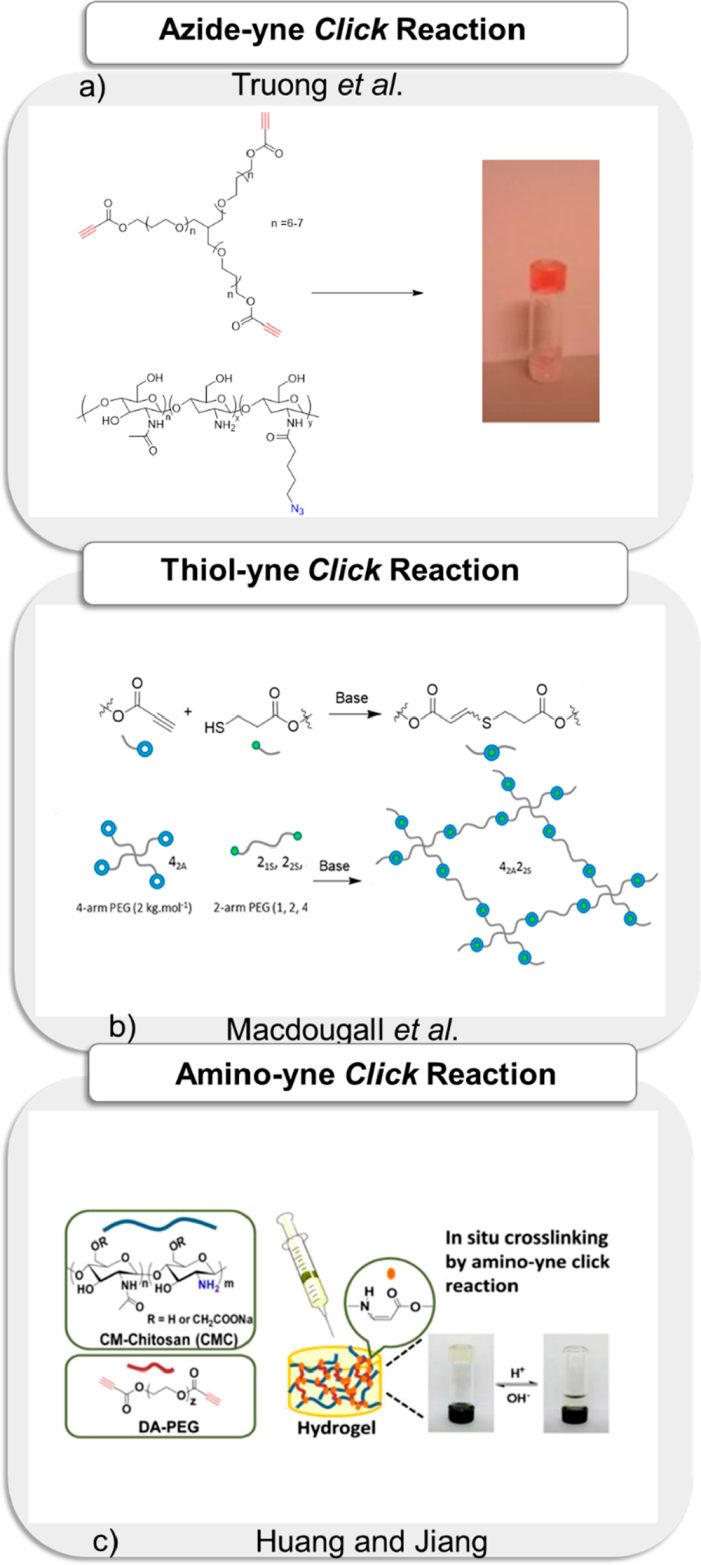
(a) Fabrication of in situ-forming chitosan-PEG hydrogels prepared by copper-free azide-alkyne click reaction. Adapted with permission from Truong et al.^222^ Copyright 2014 The Royal Society of Chemistry. (b) The fabrication of PEG-based hydrogel using nucleophilic thiol-yne “*click*” reaction. Adapted with permission from Macdougall et al.^229^ Copyright 2017 American Chemical Society. (c) The schematic representation of CMC-based hydrogels via amino-yne “*click*” reaction. Adapted with permission from Huang and Jiang.^233^ Copyright 2018 American Chemical Society.

Besides cyclooctyne, electron-deficient alkynes participate in “*click*” reaction with azide-functionalized molecules under copper-free conditions.^221−224^ This class of cycloaddition is quite attractive, since it is considerably simpler to install electron-deficient alkynes using a simple building block such as propiolic acid or its derivative. Synthesis of hydrogels using this copper-free azide-alkyne cycloaddition approach was realized as early as 2009 by Kiser and Clark, whereby they prepared gels by mixing multivalent azide-functionalized polymers with an electron-deficient bis-alkyne containing cross-linker.^221^ In a more recent work, Dove and co-workers reported fabrication of hydrogels using this metal-free cycloaddition reaction using azide-functionalized chitosan and a heterotelechelic propiolic acid ester-conjugated poly(ethylene glycol) cross-linker. The authors confirmed that the obtained hydrogel was nontoxic and supported cellular attachment (Figure 9a).^222^ In another related study, Ikeda reported the preparation of hydrogels via copper-free azide-alkyne cycloaddition reaction between azide-functionalized tetra-branched poly(ethylene glycol) and electron-deficient alkyne-functionalized tetra(ethylene glycol) in the presence of an ionic liquid, namely, 1-ethyl-3-methylimidazolium bis(trifluoromethylsulfonyl)imide.^224^ The authors claimed that the electrochemical window of the ion gel is the same as that of the ionic liquid inside the gel.

The electron-deficient alkyne functional group is also known to undergo facile addition with amine and thiol groups and thus has been recently exploited to synthesize a variety of functional polymeric materials.^225−227^ Fabrication of hydrogels using the nucleophilic thiol-yne addition reaction was reported by Xu and co-workers. PEG-based hydrogels were obtained by combining a four-arm PEGthiol (PEG_10k_-4-SH) with an electron-deficient PEG-alkyne (PEG_10k_-4-PP). After obtaining hydrogel, a thiol-containing antimicrobial peptide (AMP-SH) was incorporated into the hydrogel matrix using the same chemistry. The authors envisioned that the AMP-embedded PEG-based hydrogels exhibit low cytotoxicity against 3T3 fibroblasts and can be potentially used in wound dressing.^228^ Likewise, Dove and co-workers reported several examples of hydrogels obtained using the nucleophilic thiol-yne “*click*” reaction.^229−232^ In one of their studies, they demonstrated that this reaction is highly efficient for the fabrication of robust high-water-content hydrogels with tunable mechanical properties (Figure 9b).^229^ Another interesting variation employs the amino-yne “*click*” reaction for obtaining cross-linked materials.^233−235^ This reaction is of high interest, since several natural biopolymers possess amine groups. Huang and Jiang reported the fabrication of injectable and degradable pH-responsive carboxymethyl chitosan (CMC) hydrogels via amino-yne “*click*” reaction.^233^ They used telechelic electron-deficient dipropiolate ester of polyethylene glycol and water-soluble CMC to obtain injectable and degradable hydrogels, which were nontoxic and possessed good tissue biocompatibility (Figure 9c). The reaction was also employed by Ren and co-workers to obtain collagen-based corneal repair membranes.^234^ Langer and co-workers used this approach with synthetic polymers to obtain fast gelation in aqueous media using a multivalent secondary amine group containing small molecule cross-linkers and tetra-PEG alkynoates. The obtained hydrogels enabled cell encapsulation with >90% viability retained after 72 h.^235^ Akin to amino-yne, the aza-Michael amino-ene addition of amines to an acrylate group can also be employed to obtain cross-linked materials,^236^ and the reaction has been utilized for formulating hydrogels by Yang and co-workers, who investigated them for developing drug delivery and tissue engineering platforms.^237^

## 6 Conclusion and Perspectives

In this review, we summarized the utilization of various metal-free “*click*” reactions in fabricating hydrogels for a range of biomedical applications. While the metal-free “*click*” reactions possess attractive attributes such as high reaction efficiency, fast reaction rate, and formation of benign or no byproducts, importantly, they eliminate the limitations associated with the presence of metal catalysts. The toolbox of metal-free “*click*” reactions provides a range of effective reactions, and the choice of using a particular one may depend on factors such as ease of incorporation of the reactive handles into the hydrogel precursors, the desired rate of gelation, and its bio-orthogonal nature. As expected, all metal-free transformations have pros and cons; hence, a judicious choice should be made for choosing an appropriate one. For example, the DA reaction does not require any catalyst or photoinitiator, but the reaction may be too slow to undergo effective gelation within a short time. On the other hand, the IEDDA reactions are fast, however, the release of nitrogen gas may form bubbles, which may affect the microstructure. Likewise, while nucleophilic thiol-ene may not be entirely bio-orthogonal due to the reaction of activated alkene and thiols with several biologics, the radical thiol-ene addition requires a catalyst or photoinitiator, which can be toxic *in vivo*. Nonetheless, radical thiol-ene is excellent for various *ex vivo* applications due to high spatiotemporal control in the synthesis of microstructured hydrogels, attractive materials for tissue engineering. Likewise, the SPAAC reaction is a bio-orthogonal reaction with fast reaction kinetics and works under catalyst-free conditions with no byproduct, but the synthetic steps of cyclooctynes are complex. Despite the apparent drawbacks of some of the reactions, the rewards of using these reactions push forward by improving the individual reactions to circumvent the shortcomings. For example, the instability of the tetrazine moiety which may hamper long-term storage of hydrogel precursors does not remain of concern when tetrazine is generated *in situ* upon exposure to dihydrotetrazine under red light. This approach also provides a light-mediated “*click*” reaction like the radical thiol-ene reaction but proceeds under red light, which, unlike the commonly used ultraviolet light, does not compromise cell viability.^238^ In due course, what will be important are the solutions the “*click*”-reaction-based products will provide in tackling challenges in various arenas of biomedical sciences. Undoubtedly, a product in the clinic, coupled with an increase in clinical investigations of hydrogels fabricated using metal-free “*click*” transformations, will become the ultimate driving force in advancing this area. Looking at the current momentum in translational biomaterials research, one could only expect an increasing employment of metal-free “*click*” reactions for creating innovative multifunctional hydrogels for a variety of biomedical applications.
